# Consumption of Cross-Linked Resistant Starch (RS4_XL_) on Glucose and Insulin Responses in Humans

**DOI:** 10.1155/2010/651063

**Published:** 2009-08-23

**Authors:** Enas K. Al-Tamimi, Paul A. Seib, Brian S. Snyder, Mark D. Haub

**Affiliations:** ^1^Department of Human Nutrition, Kansas State University, Manhattan, KS 66506, USA; ^2^Department of Grain Science and Industry, Kansas State University, Manhattan, KS 66506, USA

## Abstract

*Objective*. The objective was to compare the postprandial glycemic and insulinemic responses to nutrition bars containing either cross-linked RS type 4 (RS4_XL_) or standard wheat starch in normoglycemic adults (*n* = 13; age = 27 ± 5 years; BMI = 25 ± 3 kg/m^2^). *Methods*. Volunteers completed three trials during which they consumed a glucose beverage (GLU), a puffed wheat control bar (PWB), and a bar containing cross-linked RS4 (RS4_XL_) matched for available carbohydrate content. Serial blood samples were collected over two hours and glucose and insulin concentrations were determined and the incremental area under the curve (iAUC) was calculated. *Results*. The RS4_XL_ peak glucose and insulin concentrations were lower than the GLU and PWB (*P* < .05). The iAUC for glucose and insulin were lower following ingestion of RS4 compared with the GLU and PWB trials. *Conclusions*. These data illustrate, for the first time, that directly substituting standard starch with RS4_XL_, while matched for available carbohydrates, attenuated postprandial glucose and insulin levels in humans. It remains to be determined whether this response was due to the dietary fiber and/or resistant starch aspects of the RS4_XL_ bar.

## 1. Introduction

Consumption of whole grains has been recommended to improve insulin sensitivity and lower serum glucose and insulin concentrations. Whole grain consumption of three servings or more per day was among changes that were included in the 2005 dietary guidelines to reduce the risk of acquiring chronic diseases [1]. Whole grains are major sources of dietary fiber (DF), yet typical DF consumption patterns do not meet the recommended 25–35 g per day. Therefore, eating more grain-based fiber-rich foods is warranted to help optimize health and potentially manage some chronic metabolic conditions.

At present, resistant starches (RSs) have drawn broad interest for their health benefits and functional properties [2, 3]. Initial clinical studies demonstrated that RS has properties similar to soluble fiber, shows promising physiological benefits in humans, and may prevent disease. Several potential physiological benefits ascribed to RS include attenuation of blood glucose and insulin levels in both healthy and diabetic individuals, positive effects on large bowel health and prevention of colonic cancer, increased absorption of minerals, serving as a prebiotic, and increased fat oxidation [3–9]. There are four basic “types” of RS. Type 1 (RS1) is composed of starch granules embedded in indigestible plant material. Type 2 (RS2) is native granular starch with a B-type x-ray pattern, such as found in potato and high-amylose maize. Type 3 (RS3) is crystallized starch and maltodextrins made by alternate cooking/cooling processes on starchy materials. Type 4 (RS4) is chemically modified starch typically through esterification, crosslinking (RS4_XL_), or transglycosylation. 

The majority of human clinical trials have been conducted using only RS2 or RS3, which tend to illustrate decreased blood glucose following consumption of foods with these starches added [3, 6, 10–15]. It is difficult to fully understand the beneficial capacity of RS due to the methods used in the human clinical trials that tested the efficacy of RS. For example, one clinical trial failed to control both the amount and source of all the ingredients [16]. In a study investigating the effects of esterified RS type 4 (RS4_OSA_), Heacock et al. [17] reported that RS4_OSA_ decreased peak glucose and insulin levels when it was administered in water, but RS is typically consumed in foods. Also, in studies by Behall et al. [18, 19], the amount of available carbohydrate differed, which limits the capacity to determine if the attenuation of glucose and insulin was due to the RS or the fact there was less available carbohydrate. Furthermore, Robertson et al. [6] provided packets containing RS2 for volunteers to sprinkle on their food thereby not illustrating the effects that might be achieved when provided in the food supply. Taken together, the available data illustrate that RS has the potential to lower blood glucose. However, few clinical trials testing the effects of RS have controlled ingredients and the amount of available carbohydrates to better delineate the role of RS in affecting the insulin and glucose responses, and no published clinical trials investigating the glucose lowering potential of RS4_XL_ exist. 

Therefore, the aim of this study was to investigate the acute effects of consuming RS4_XL_ incorporated into a nutrition bar, while controlling for nonstarch ingredients and available carbohydrates, on postprandial glucose and insulin responses in young adults with a randomized clinical trial (NCT00687960, clinicaltrials.gov).

## 2. Materials and Methods

### 2.1. Subjects

The Institutional Review Board of Kansas State University approved the study, and written informed consent was obtained from all volunteers prior of the study. Inclusion criteria were no diagnosis of acute or chronic metabolic diseases, free of gastrointestinal disorders, body mass index of 23–30 kg/m^2^, and nonsmokers. Volunteers were screened for glucose tolerance using a two hour 75 g glucose tolerance test prior to participation. Eighteen healthy younger adults were recruited and 13 participated in and completed the study, while the other five did not meet the criteria. Of the 13 volunteers (age = 27 ± 5 years, BMI = 25 ± 3 kg/m^2^, HOMA = 0.94 ± 0.34), 7 were females and 6 were males. Based on BMI values, these volunteers all have similar risks for all-cause and obesity-related mortality [20]. Also, an oral glucose tolerance test was performed prior to enrollment to ensure each volunteer had normal glucose tolerance.

### 2.2. Oral Glucose Tolerance Test

Prior to enrollment, volunteers arrived after a 12 hours overnight fast. Blood samples were drawn by finger stick at baseline and 120 minutes after ingesting 75 g of glucose in solution (296 mL; Sun-Dex 75 g, Fisher Scientific, Houston, Tex, USA). Samples were analyzed in duplicate for glucose concentration (YSI 2300 STAT, Yellow Springs, Ohio, USA). The oral glucose tolerance test was used to confirm the absence of prediabetes or diabetes.

### 2.3. Study Design

All trials were performed at the Human Metabolism Laboratory at Kansas State University. Volunteers completed three trials via a controlled randomized crossover design. During each trial, volunteers consumed one of the following: dextrose solution (198 mL of a standard 75 g oral glucose tolerance beverage; GLU), a control bar containing puffed wheat (65 g; PWB), and a bar containing cross-linked RS4_XL_ bar (80 g; RS4_XL_) (Table 1). All treatments were designed to provide 50 g of available carbohydrate (Table 2). All trials were completed by each volunteer with at least a seven-day washout between testing days. This was a quasiblinded experiment in that one treatment was a beverage and the other two were in bar form, which were randomly administered using a Latin Square design. Some female volunteers did not use oral contraceptives, but others used either contraceptive pills or progesterone injections. Regardless, the females were scheduled to perform all the trials during the follicular phase of their menstrual cycle and each served as their own control.

### 2.4. Experimental Bars

The only altered ingredients between bars were either puffed wheat or RS4_XL_ (Table 1). Briefly, the nutrition bars were prepared by adding puffed wheat or cross-linked RS4 to wheat germ. The dose of the RS4_XL_ (27.2 g) was intended to be close to the dose used previously [6], whereby a significant improvement in insulin sensitivity was observed. The remaining ingredients (water, corn syrup, brown sugar, gum acacia, and Panodan 150 K) were heated to 85°C over 4 minutes, poured over the dry ingredients, and then manually mixed quickly until dry ingredients were evenly distributed throughout the mixture. The mixture was scooped into a metal pan, pressed evenly throughout the pan, and allowed to cool for 20 minutes before cutting into bars. Crude nutrient analysis was determined by proximate analysis, while total dietary fiber was assessed independently (Medallion Labs, Minneapolis, Minn) (Table 2). Available carbohydrate was calculated as the difference between total carbohydrate and dietary fiber as used previously [9]. Controlling for available carbohydrate in this fashion has been shown to affect the glycemic response, while controlling for RS content does not necessarily affect the glycemic response [21].

### 2.5. Food Tolerance Test

The postprandial test (two-hours with seven blood samples) was modified from Flammang et al. [22]. During each test, volunteers arrived to the laboratory after a 10–12 hour overnight fast. An indwelling catheter (Terumo, 22gx1, Terumo Medical Corporation, Elekton, Md) was inserted into a forearm vein. The line was kept patent with 0.9% isotonic saline solution (0.9% sodium chloride USP, B. Braun Medical Inc., Irvine, Calif). Ten minutes after inserting the IV catheter, the fasting blood sample was collected. Thereafter, the volunteers consumed the assigned food item for that day. The solution (GLU) or bar was consumed within 10 minutes. The treatment bars were served with 198 mL of water (to match the fluid that was consumed during the GLU trial). Relative to taking the first bite of food, blood samples were collected at −10, 10, 20, 30, 60, 90, and 120 minutes.

### 2.6. Blood Samples

After collection, blood samples were centrifuged at 2,500 rpm for 15  minutes at 4°C. The plasma was extracted from the vacutainer and immediately analyzed in duplicate for glucose (YSI 2300, Yellow Springs Instruments, Yellow Springs, Ohio) with the remainder stored at −80°C. The frozen samples were analyzed in duplicate for insulin using an endocrine assay (LINCOplex kit, St. Charles, Mo), and measured by Luminex100 (Austin, Tex) instrumentation. Once the samples were analyzed, the highest value attained over the 120 minutes was determined to be the peak value, with the difference between baseline and peak determined to measure the change from fasting to peak.

Glucose and insulin areas under the curve (iAUC) were determined using the trapezoid method (GraphPad Prism v 5.02, La Jolla, Calif). This approach was adapted from a previous RS feeding study [18]. Fasting insulin and glucose values were also used to calculate the homeostasis model assessment (HOMA) [23].

### 2.7. Diet and Physical Activity Records

A diet record was completed by the volunteers prior the first test of the study. Volunteers were instructed on how to record their intakes. They were requested to then eat the same foods from that diet recorded the day before the second and third trials. This procedure was used previously [24]. Also, volunteers were requested to record their physical activity for the day before testing and perform that same activity (or inactivity) the day prior to the subsequent tests.

## 3. Data Analysis

Sample size estimation was calculated using PASS software (NCSS 2007 and PASS 2005, Kaysville, Utah). Based on results from previously published data using RS2 [6], six volunteers were determined necessary (power >0.80, and *P* < .05) to detect significant differences in glucose and insulin responses. A repeated measures analysis of variance (SPSS version 11.5, Chicago, Ill) was used to determine significant main effects with significance set at *P* = .05. Paired *t*-tests (SPSS version 11.5, Chicago, Ill) were used to determine differences between and among trials for peak, change from baseline to peak and iAUC values for glucose and insulin. The comparisons of interest were primarily between the two bars, with the glucose treatment providing a standard point of reference.

## 4. Results

The three meals provided practically the same amount (50-51 g) of available carbohydrate (Table 2). Both nutrition bars contained similar amounts of protein and were low in fat, but the RS4_XL_ bar contained four times the dietary fiber (20 g versus 5 g) compared to the PWB. Approximately one-fifth of the total dietary fiber in the RS4_XL_ bar was from gum arabic (~4.4 g) with the remainder (~15.6 g) being from RS4_XL_. The meals differed in their calculated food-energy contents (200–326 kcal, Table 2) because their compositions differed and because their weights to deliver 50 g of available carbohydrates differed.

The commercially available RS4_XL_ used in this study contains 0.4% phosphors, 10.6% moisture, 91.9% total dietary fiber by AOAC-International Method 991.43, and 83.3% RS by a modified Englyst method [25]. At the time of preparing the 80 g bar of RS4_XL_, the amount of RS added was ~20 g (dry solids) according to the formula in Table 1 and the composition of Fibersym RW, while the amount of gum arabic added was 4.4 g (dry solids). At the time of analyzing the finished 80 g bar, the bar contained 20 g of total dietary fiber (Table 2). Assuming no loss of gum arabic in the preparation of the RS4_XL_ bar, the 4.4 g loss of dietary fiber could be attributed to some damage to the RS, which increased its digestibility. Assuming the ratio of RS to dietary fiber remains constant at 0.9 in partially damaged RS4_XL_, then the 80 g bar of RS4_XL_ fed to the subjects contained ~14 g RS, implying ~70% was maintained following preparation of the bar.

**Figure 1:**
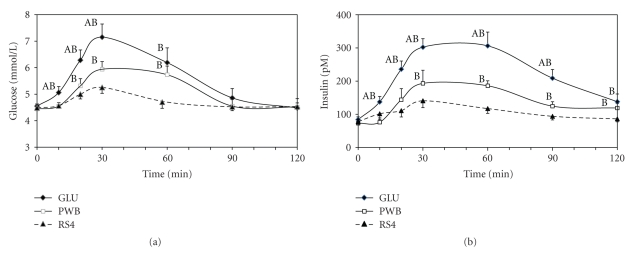
Depiction the glucose (a) and insulin (b) responses over two hours following the consumption of each (Glu, PWB, and RS4) treatment. Values represent each mean ± SE. A: significant difference with PWB; B: significant difference with RS4.

The RS4 bar elicited decreased glucose and insulin concentrations at several time points (Figure 1) during the 120 minutes postprandial period as compared with GLU and PWB. Also, consumption of the RS4 bar led to an attenuation of peak glucose and insulin, and significant differences from baseline to peak and iAUC values glucose and insulin when compared with GLU and PWB (Table 3). The PWB bar attenuated the peak glucose and insulin responses and the iAUC for glucose and insulin compared with GLU. The percent increase from baseline to peak was not different (*P* = .068) from GLU for glucose, while it was for insulin.

## 5. Discussion

These data, for the first time, indicate that eating RS4_XL_ from wheat in place of standard wheat starch significantly decreased postprandial insulin and glucose responses. These results are in line with others investigating the insulin and/or glucose lowering effects when RS (typically RS2) is added to foods or incorporated in the diet [6, 16, 18, 26, 27], while a few reported no effect of RS2 or RS3 on glycemia [7, 9]. Results from several other clinical trials reported RS decreased the glycemic response, but those studies had volunteers sprinkle RS onto the food instead of it being an ingredient in the food, mixed only with water, ate large (up to 388 g) portion sizes, failed to control for available carbohydrate, and/or the food eaten contained different ingredients with varying amounts of available carbohydrate [6, 16, 18, 26, 27].

One of the more surprising outcomes was that RS4_XL_ significantly attenuated the glycemic and insulinemic responses even when high glycemic ingredients were eaten. This observation is critical for consumers and food scientists when looking for or creating foods to control blood glucose levels in that they may not need to avoid foods containing corn syrup or sugar for the purpose of regulating their blood glucose levels. That is, these data clearly indicate that even though two foods contain identical concentrations of glycemic ingredients, the presence of RS4_XL_ may significantly lower glucose and insulin responses. In the case of the bars that were tested, the volunteers actually consumed more corn syrup and brown sugar in the RS4_XL_ bar since they consumed 15 g more of that bar than the PWB bar. Yet, the glycemic and insulinemic responses for the RS4_XL_ bar were significantly attenuated compared with the PWB bar that was matched for available carbohydrate yet contained less (by weight) sugar and corn syrup (Table 3). This does not imply that eating sugar and corn syrup is healthy, but that foods high in RS4_XL_ attenuate glycemia and insulinemia compared with those with standard whole wheat starch when sugar and corn syrup are present and account for ~30% of the energy. This is of interest as sugar and corn syrup have been the target of criticism for potentially contributing to the obesity and diabetes epidemics [28–30]. 

The observed attenuation of the glucose and insulin responses might be a result of high concentrations of DF and/or RS in the RS4_XL_ bar. In separate in vitro analyses for DF and RS, this version of cross-linked RS4 has been shown to contain 91.9% dietary fiber and 83% resistant starch [25]. Relative to clinical trials that used RS2, Le Leu et al. [31] recently reported that several varieties of RS2 only contain 18–60% dietary fiber and 46–53% RS. Thus, this cross-linked form of RS4 likely elicited the significant difference due to a relatively high content of fiber and RS.

This study is not without limitations. While we did observe a significant attenuation in glucose and insulin responses when RS4_XL_ was incorporated into the bar, it is not possible to know whether the same effects would occur in individuals with insulin resistance or other metabolic conditions. It could be suggested that the current dose used for the RS4_XL_ bar (80 g) might be greater than one would choose to consume at one sitting. A more realistic approach might be to base the doses on average serving sizes, but that would only offer 15–20 g of available carbohydrate in the RS4_XL_ bar. That said a previous study [26] had volunteers consume treatment foods that were nearly five-fold greater. Likewise, we were trying to match the amount of available carbohydrate at 50 g, which is recommended for glycemic index testing [32]. Another potential confounding issue, especially when comparing the bars with the solution, is the difference in gastric emptying [33]. Also, the total energy contained within the RS4_XL_ bar did not take into account the energy provided via fermentation of the RS_XL_ into short chain fatty acids. Lastly, we cannot determine the mechanism for the effect observed. It is reasonable to conclude that the RS4_XL_ caused the effects, as it was the only ingredient difference, but it is not possible to determine how much of the change was attributable to the dietary fiber or the RS that is contained within RS4_XL_.

In conclusion, this is the first published randomized clinical trial to investigate the glucose and insulin lowering potential of RS4_XL_. Additionally, this is one of a few clinical studies where the treatments were matched for available carbohydrate and the RS was substituted directly for standard starch in the tested food, while all other ingredients were identical. This ingredient and nutrient control minimizes confounding factors that are present when treatments are matched instead for total carbohydrate [18] or when different ingredients are used in clinical trials [16]. While it is unknown what component (dietary fiber or resistant starch) of RS4_XL_ lowered glucose and insulin, it is clear that RS4_XL_ was responsible for the observed differences between bars. 

## Figures and Tables

**Table 1:** Ingredients and their concentrations by relative weight (% total) in the test bars.

	PWB	RS4_XL_
Puffed Wheat^a^	34	—
Resistant Starch type 4^b^	—	34
Corn Syrup^c^	20	20
Wheat Germ^d^	18	18
Brown Sugar^e^	11	11
Water^f^	10	10
Gum Acacia^g^	6	6
Panodan 150K^h^	1	1

^a^ Quaker Oats

^b^ Fibersym RW; MGP Ingredients, Inc.

^c^ Karo light corn syrup

^d^ Kretschmer Original Toasted

^e^ C&H Pure Cane Sugar, golden brown

^f^ Tap water (Manhattan, Kan)

^g^ TIC Gums

^h^ Danisco

**Table 2:** Nutrient composition of each treatment per dose (GLU = 198 mL; PWB = 65 g; RS4 = 80 g).

	GLU^a^	PWB^b^	RS4_XL_^b^	Δ^e^
Total Energy (kcal)	200	261	326	(65 kcal, 125%)
Carbohydrate (g)				
Total	50	56	71	(15 g, 127%)
Available^c^	50	51	51	(0 g, 0%)
Total Dietary Fiber (g)^d^	—	5	20	(15 g, 400%)
Fat (g)	—	1	2	(1 g, 200%)
Protein (g)	—	7	6	(1 g, 86%)

^a^ glucose tolerance test beverage (Sun-Dex, Fisher Scientific, Houston, Tex)

^b^ Crude nutrient composition was determined by proximate analysis (total energy, total fat, total protein, total carbohydrate).

^c^ derived by subtracting total dietary fiber from total carbohydrate.

^d^ dietary fiber analysis was performed by Medallion Laboratory (Minneapolis, Minn).

^e^ difference (subtraction value, % value) between bars.

**Table 3:** Values for the incremental areas under the curves of glucose and insulin concentrations during each trial00. Mean ± SE; different letters within a row indicates significant difference (*P* < .05).

	GLUC	PWB	RS4_XL_
Glucose			
iAUC (mmol/L • 2 hr)	140 ± 31^A^	84 ± 17^B^	28 ± 11^C^
Peak (mmol/L)	7.30 ± 0.5^A^	6.33 ± 0.3^B^	5.40 ± 0.2^C^
Increase (%)	60.5 ± 10^A^	42.7 ± 6^A^	20.4 ± 3^B^

iAUC (pM • 2 hr)	17,575 ± 2,236^A^	8,758 ± 1,132^B^	3,659 ± 974^C^
Peak (pM)	344 ± 36.7^A^	211.5 ± 20.1^B^	162.3 ± 22.6^C^
Increase (%)	335 ± 53.2^A^	243.0 ± 49.3^B^	126.3 ± 45.8^C^

## Abbreviations

DF: Dietary fiber
GLU: Glucose control solution
iAUC: Incremental area under the curve
HOMA: Homeostasis Model Assessment of insulin sensitivity
PWB: Control bar made with puffed wheat
RS: Resistant starch
RS4_XL_: Treatment bar made with cross-linked resistant starch type 4.

## References

[1] U.S. Department of Health and Human Services, U.S. Department of Agriculture (2005). *Dietary Guidelines for Americans*.

[2] Higgins JA (2004). Resistant starch: metabolic effects and potential health benefits. *Journal of AOAC International*.

[3] Jenkins DJA, Kendall CWC (2000). Resistant starches. *Current Opinion in Gastroenterology*.

[4] Zhou J, Martin RJ, Tulley RT (2008). Dietary resistant starch upregulates total GLP-1 and PYY in a sustained day-long manner through fermentation in rodents. *American Journal of Physiology*.

[5] So P-W, Yu W-S, Kuo Y-T (2007). Impact of resistant starch on body fat patterning and central appetite regulation. *PLoS ONE*.

[6] Robertson MD, Bickerton AS, Dennis AL, Vidal H, Frayn KN (2005). Insulin-sensitizing effects of dietary resistant starch and effects on skeletal muscle and adipose tissue metabolism. *American Journal of Clinical Nutrition*.

[7] Higgins JA, Higbee DR, Donahoo WT, Brown IL, Bell ML, Bessesen DH (2004). Resistant starch consumption promotes lipid oxidation. *Nutrition & Metabolism*.

[8] Topping DL, Fukushima M, Bird AR (2003). Resistant starch as a prebiotic and synbiotic: state of the art. *Proceedings of the Nutrition Society*.

[9] Jenkins DJA, Vuksan V, Kendall CWC (1998). Physiological effects of resistant starches on fecal bulk, short chain fatty acids, blood lipids and glycemic index. *Journal of the American College of Nutrition*.

[10] Yamada Y, Hosoya S, Nishimura S (2005). Effect of bread containing resistant starch on postprandial blood glucose levels in humans. *Bioscience, Biotechnology and Biochemistry*.

[11] Akerberg A, Liljeberg H, Bjorck I (1998). Effects of amylose/amylopectin ratio and baking conditions on resistant starch formation and glycaemic indices. *Journal of Cereal Science*.

[12] Bjorck I, Liljeberg H, Ostman E (2000). Low glycaemic-index foods. *British Journal of Nutrition*.

[13] Vonk RJ, Hagedoorn RE, de Graaff R (2000). Digestion of so-called resistant starch sources in the human small intestine. *American Journal of Clinical Nutrition*.

[14] Hoebler C, Karinthi A, Chiron H, Champ M, Barry J-L (1999). Bioavailability of starch in bread rich in amylose: metabolic responses in healthy subjects and starch structure. *European Journal of Clinical Nutrition*.

[15] Witwer RS, Pasupuleti VK, Anderson JW (2008). Natural resistant starch in glycemic management; from physiological mecanisms to consumer communications. *Nutraceuticals Glycemic Health, and Type 2 Diabetes*.

[16] Reader DM, O’Connell BS, Johnson ML, Franz M (2002). Glycemic and insulinemic response of subjects with type 2 diabetes after consumption of three energy bars. *Journal of the American Dietetic Association*.

[17] Heacock PM, Hertzler SR, Wolf B (2004). The glycemic, insulinemic, and breath hydrogen responses in humans to a food starch esterified by 1-octenyl succinic anhydride. *Nutrition Research*.

[18] Behall KM, Scholfield DJ, Hallfrisch JG, Liljeberg-Elmstahl HG (2006). Consumption of both resistant starch and *β*-glucan improves postprandial plasma glucose and insulin in women. *Diabetes Care*.

[19] Behall KM, Scholfield DJ, van der Sluijs AMC, Hallfrisch J (1998). Breath hydrogen and methane expiration in men and women after oat extract consumption. *Journal of Nutrition*.

[20] Flegal KM, Graubard BI (2009). Estimates of excess deaths associated with body mass index and other anthropometric variables. *American Journal of Clinical Nutrition*.

[21] Granfeldt Y, Wu X, Bjorck I (2006). Determination of glycaemic index; some methodological aspects related to the analysis of carbohydrate load and characteristics of the previous evening meal. *European Journal of Clinical Nutrition*.

[22] Flammang AM, Kendall DM, Baumgartner CJ, Slagle TD, Choe YS (2006). Effect of a viscous fiber bar on postprandial glycemia in subjects with type 2 diabetes. *Journal of the American College of Nutrition*.

[23] Matthews DR, Hosker JP, Rudenski AS (1985). Homeostasis model assessment: insulin resistance and *β*-cell function from fasting plasma glucose and insulin concentrations in man. *Diabetologia*.

[24] Haub MD, Potteiger JA, Jacobsen DJ, Nau KL, Magee LA, Comeau MJ (1999). Glycogen replenishment and repeated maximal effort exercise: effect of liquid carbohydrate. *International Journal of Sport Nutrition*.

[25] Yeo LL, Seib PA (2009). White pan bread and sugar-snap cookies containing wheat starch phosphate, a cross-linked resistant starch. *Cereal Chemistry*.

[26] Nilsson AC, Ostman EM, Holst JJ, Bjorck IME (2008). Including indigestible carbohydrates in the evening meal of healthy subjects improves glucose tolerance, lowers inflammatory markers, and increases satiety after a subsequent standardized breakfast. *Journal of Nutrition*.

[27] Raben A, Tagliabue A, Christensen NJ, Madsen J, Holst JJ, Astrup A (1994). Resistant starch: the effect on postprandial glycemia, hormonal response, and satiety. *American Journal of Clinical Nutrition*.

[28] Gross LS, Li L, Ford ES, Liu S (2004). Increased consumption of refined carbohydrates and the epidemic of type 2 diabetes in the United States: an ecologic assessment. *American Journal of Clinical Nutrition*.

[29] Basciano H, Federico L, Adeli K (2005). Fructose, insulin resistance, and metabolic dyslipidemia. *Nutrition & Metabolism*.

[30] Bray GA, Nielsen SJ, Popkin BM (2004). Consumption of high-fructose corn syrup in beverages may play a role in the epidemic of obesity. *American Journal of Clinical Nutrition*.

[31] Le Leu RK, Hu Y, Brown IL, Young GP (2009). Effect of high amylose maize starches on colonic fermentation and apoptotic response to DNA-damage in the colon of rats. *Nutrition & Metabolism*.

[32] Jenkins DJA, Wolever TMS, Taylor RH (1981). Glycemic index of foods: a physiological basis for carbohydrate exchange. *American Journal of Clinical Nutrition*.

[33] Livesey G, Tagami H (2009). Interventions to lower the glycemic response to carbohydrate foods with a low-viscosity fiber (resistant maltodextrin): meta-analysis of randomized controlled trials. *American Journal of Clinical Nutrition*.

